# 85 A synthesis of the qualitative evidence exploring participants’ experiences of intergenerational dance programmes

**DOI:** 10.1093/eurpub/ckae114.145

**Published:** 2024-09-26

**Authors:** Siobhán O’Reilly, Orfhlaith Ní Bhriain, Sarah Dillon, Amanda Clifford

**Affiliations:** School of Allied Health, University of Limerick, Castletroy, Ireland; Health Research Institute, University of Limerick, Ireland; Ageing Research Centre, University of Limerick, Ireland; Health Research Institute, University of Limerick, Ireland; Irish World Academy of Music and Dance, University of Limerick, Castletroy, Ireland; School of Allied Health, University of Limerick, Castletroy, Ireland; Health Research Institute, University of Limerick, Ireland; Ageing Research Centre, University of Limerick, Ireland; School of Allied Health, University of Limerick, Castletroy, Ireland; Health Research Institute, University of Limerick, Ireland; Ageing Research Centre, University of Limerick, Ireland

## Abstract

**Purpose:**

The aim of this review was to synthesise the qualitative evidence that explored participants’ experiences in intergenerational dance programmes. Low levels of physical activity (PA) and loneliness are prevalent concerns affecting physical and emotional health across the lifespan. Dance interventions can improve health and reduce loneliness by creating a sense of belonging. Intergenerational interventions can create bonds and improve relationships between older and younger cohorts. To date, no qualitative review has specifically explored experiences of people who participated in intergenerational dance programmes.

**Methods:**

Nine interdisciplinary databases were searched. The inclusion criteria were qualitative or mixed-methods studies that explored participants’ experiences of intergenerational dance. Exclusion criteria were dance movement therapy and no generational gap. Thomas and Harden’s (2008) method of thematic synthesis was used to analyse data. Five papers were coded using NVivo and a coding tree was generated, with descriptive themes identified. The quality of papers was assessed using the Critical Appraisal Skills Programme.

**Results:**

Five papers were included in the analysis, three qualitative and two mixed-methods (n = 133). Dance genre was not specified, however all involved intergenerational activity. Two themes were identified. Theme one was Socialisation, Communication and Participant Connections. An experience for both cohorts was relationship building. Loneliness was identified as a motivating factor for participation in older adults, with some participants feeling more connected to their community afterwards. The second theme was A Beneficial Experience. Older adults felt positive emotions towards the programmes. Self-reported health improvements were highlighted such as staying out of the doctor’s office. Caregivers noticed improvements in participants. Older adults found that communication with students helped build support systems. Younger people gained a greater understanding of older adults.

**Conclusions:**

Intergenerational dance programmes were found to be a valuable way to connect with communities. Both older adults and young people have found the interventions enjoyable and beneficial. Loneliness has been linked to increased physician visits, and this review shows emerging evidence to suggest that these programmes can mitigate this, which may reduce overall healthcare costs. These programmes should be considered as viable methods to encourage PA and socialisation within communities.

**Funding:**

Irish Research Council GOIPG/2023/4704

